# The Multisensory Hallucinations Schedule to assess hallucinations across multiple sensory modalities in psychosis: A pilot validation study

**DOI:** 10.1177/00048674261457016

**Published:** 2026-06-23

**Authors:** Wei Lin Toh, Adrienne Bell, Mikaela Bere, Susan Lee Rossell

**Affiliations:** 1 Centre for Mental Health & Brain Sciences, Swinburne University of Technology, Melbourne, VIC, Australia; 2 Department of Psychology, Alfred Hospital, Melbourne, VIC, Australia; 3 Department of Psychiatry, St Vincent’s Hospital, Melbourne, VIC, Australia; 4 InsideOut Institute, University of Sydney, Sydney Local Health District, Sydney, NSW, Australia

**Keywords:** Psychosis, multisensory/multimodal, delusions, scale validation, assessment

## Abstract

**Objective:**

Hallucination studies in psychiatry have typically focused on the auditory domain, despite mounting evidence that non-auditory hallucinations are prevalent, and elicit distress and functional interference. Proponents have called for more in-depth phenomenological enquiries into hallucinations across multiple sensory modalities, but there are no comprehensive, validated assessment tools to date. This study aimed to document the design and pilot validation of such a transdiagnostic instrument – the Multisensory Hallucinations Schedule.

**Methods:**

Leveraging off a precursor measure, the Multisensory Hallucinations Schedule comprised eight modules: *Auditory*, *Visual*, *Olfactory*, *Somatic-tactile*, *Gustatory*, *Other modalities*, *Multimodal* and *Delusions*, across lifetime and current time frames. Pilot validation analyses sought to confirm its factor structure via principal components analyses, verify acceptability, convergent and divergent validities, and compute reliability (equivalence and stability) statistics as well as internal consistencies.

**Results:**

Based on 76 participants diagnosed with a primary psychotic illness, the Multisensory Hallucinations Schedule demonstrated acceptable factor structure, with two- to four-factor solutions generated across its subscales, explaining 39.2–76.9% of variance. It displayed excellent acceptability (98.7% completion) as well as robust convergent (*r* < 0.807) and divergent (*r* > 0.005) validities. Moderate to high equivalence (intraclass correlation coefficient = 0.574–0.993) and stability (intraclass correlation coefficient = 0.535–0.991), and good internal consistency (Cronbach’s α = 0.462–0.851) were shown.

**Conclusions:**

The Multisensory Hallucinations Schedule represents an initial foray into inclusively documenting multisensory hallucinations in psychosis. Future validation endeavours should look towards replication using more robust participant numbers as well as expanding to other psychiatric and neurological cohorts experiencing prominent hallucinations.

## Introduction

A preponderance of research has centred on auditory hallucinations within psychotic illness, likely owing to its elevated endorsement across disorders as well as concomitant distress and functional impairment (e.g. Larøi et al., 2012; Waters et al., 2012). The study of visual hallucinations has been somewhat relegated to neurological disorders (e.g. O’Brien et al., 2020), whereas lesser known domains, involving olfactory, somatic-tactile, gustatory or other modalities, have largely been overlooked. Beyond examining their presence, researchers have been calling for more in-depth phenomenological studies to gain clarity regarding the characteristics of these experiences (Johns et al., 2014; Upthegrove et al., 2016). The clinical relevance of this endeavour would be to inform the development of effective interventions, for when associated distress is evidenced. But to do so, we first need to be able to comprehensively assess these multisensory phenomena in a systematic manner to gather useful information regarding their prevalence and impact, among other characteristics of interest.

Limited studies in psychiatry have focused on the primacy of non-auditory hallucinations, with a few exceptions (Cachia et al., 2020; Chouinard et al., 2019; Dudley et al., 2019; Lewandowski et al., 2009; Stevenson et al., 2011). Of late, research attention has highlighted the significance of hallucinations occurring in two or more sensory modalities, known as multisensory hallucinations (McCarthy-Jones et al., 2017; Montagnese et al., 2020). When they occur concurrently in time, and/or share thematic overlaps, use of the term multimodal hallucinations has been suggested to distinguish these experiences (Toh et al., 2021). Notably, an approach involving unbiased enquiry of common sensory domains and their phenomenological correlates has rarely been attempted. A major obstacle hindering these endeavours is the lack of comprehensive, validated instruments (Montagnese et al., 2020; Toh et al., 2021). The current paper thus aimed to document the design and validation of such a tool to advance the field of hallucination research.

### Existing measures of hallucinations and delusions

Majority of established psychosis measures offer excellent utility, validity and reliability, but are often circumscribed to only assess specific symptoms of interest. For instance, the Psychotic Symptom Rating Scales (PSYRATS; Haddock et al., 1999) taps into phenomenological aspects of auditory hallucinations and the impact of delusions, but neglects to inquire about other sensory modalities. Likewise, the Scale for the Assessment of Positive Symptoms (SAPS; Andreasen, 1984) documents presence, but not phenomenology, of hallucinations across several dominant senses (although it overlooks gustatory), as well as a range of delusional themes. The most comprehensive phenomenological examination of hallucinations to date is offered by the Mental Health Research Institute Unusual Perceptions Schedule (MUPS; Carter et al., 1995), but again, focus is on the auditory domain, although other modalities (occurring on the periphery of voice-hearing) are touched on. However, its meticulousness and concomitant lengthy administration may serve as a deterrent in time-limited research environments. Assessments of visual hallucinations tend to derive from neurological research, where for example, the North-East Visual Hallucinations Interview (NEVHI; Mosimann et al., 2008) offers a robust evaluation of these experiences and related phenomenological facets, although again omitting other sensory modes. Validated hallucination measures in other sensory modalities (e.g. olfactory) are generally lacking in the field.

Some novel measures of hallucinations across multiple sensory domains have more recently emerged, including the Psycho-Sensory hAllucinations Scale (PSAS; de Chazeron et al., 2015), Multi-modality Unusual Sensory Experiences Questionnaire (MUSEQ; Mitchell et al., 2017) and Questionnaire for Psychotic Experiences (QPE; Rossell et al., 2019). The PSAS considers auditory, visual, olfactory-gustatory and cenesthetic hallucinations, with four modules comprising largely corresponding phenomenological questions tapping into *frequency*, *duration*, *negative valance*, *conviction*, *impact* and *controllability*, rated on 5-point Likert-type scales. There are also dichotomous questions at the start of each module gauging the nature, complexity and origin of hallucinations in the sensory modes under consideration. The PSAS has been validated transdiagnostically in schizophrenia and Parkinson’s disease (de Chazeron et al., 2015) and offers utility for phenomenological examination of hallucinations in these four sensory modalities (albeit combining olfactory and gustatory), but with no consideration of multisensory/multimodal interactions or delusions.

The MUSEQ taps into a myriad of unusual sensory experiences segregated by domain (*auditory*, *visual*, *olfactory*, *gustatory*, *bodily sensations*, *sensed presence*), rated on 5-point Likert-type scales, but only calibrated for *frequency*. It provides an inclusive snapshot across multiple sensory domains within a succinct administration time frame, but at the expense of loss of phenomenological detail, and neglect of multisensory/multimodal interactions and delusions (like the PSAS). Its self-report format also raises questions regarding accuracy of information collected, given the complex nature of these phenomena as well as lack of corroboration and clinical judgement afforded by face-to-face interviews.

The QPE has shown good promise and comprises four modules evaluating *auditory*, *visual* and *hallucinations in other modalities*, as well as *delusions*. The *auditory* and *visual* modules assess comprehensive phenomenological features in parallel, whereas the *delusions* module similarly taps into a balanced mix of delusional themes and related impact. The *hallucinations in other modalities* module, however, only offers a cursory touch on olfactory, somatic-tactile and multisensory hallucinations as well as sensed presence, with lesser phenomenological detail (only a single frequency and/or description question per construct). Other modes of hallucinations, for instance involving gustatory or hypnagogic-hypnopompic hallucinations, are also missing. While the QPE has undergone large-scale transdiagnostic validation in psychosis samples (Rossell et al., 2019), further enhancements may improve its utility and range of assessment.

### Aims of the current study

The current study aimed to address an identified gap in knowledge by describing the design and pilot validation of a comprehensive multisensory hallucinations and delusions measure – the Multisensory Hallucinations Schedule (MHS). First, we outlined the design process underlying the MHS. Second, we embarked on preliminary validation of this instrument, involving: (1) confirmation of the factor structures of its subscales, (2) corroboration of acceptability, convergent and discriminant validities and (3) verification of interrater and test–retest reliabilities as well as internal consistencies.

## Method

### Participants and procedure

Participants were recruited from outpatient services at St Vincent’s Hospital and the Alfred Hospital, as well as physical and online advertising at community support groups and research participation forums in Melbourne, Australia. To be eligible, prospective participants had to have a primary psychosis-related diagnosis (e.g. schizophrenia spectrum disorder or mood disorder with psychotic features) and endorse hallucinatory experiences in one or more sensory modalities on at least three distinct occasions over their lifetime. No specific psychiatric disorders were targeted (although primary diagnosis was recorded). These diagnoses were derived via the Mini International Neuropsychiatric Interview (MINI; Sheehan et al., 1998) deployed as a screening tool (*n* = 55), or as diagnosed by their primary treating clinician (*n* = 21). To maximise representativeness, common psychiatric (e.g. anxiety and depressive disorders) and neurological (e.g. autism spectrum disorders, epilepsy and migraine) comorbidities were permitted; significant neurodegenerative disorders (e.g. Huntington’s or Parkinson’s disease) and traumatic brain injury were excluded. The following inclusion criteria also applied: (1) aged ⩾ 18 years, (2) fluent speaker of the English language, (3) no current substance use disorder in the past 12 months, (4) stable type and dose of antipsychotic medication in the past 8 weeks and (5) no electroconvulsive therapy in the past 6 months.

Measures (see section ‘Materials’) were administered in the same order, and all raters (A.B., C.Y., M.B.) were qualified research associates or higher degree students, trained to administer assessments in a standardised manner. A subset of participants (*n* = 21; ~30%) underwent a second MHS assessment to collect ratings for interrater and test–retest reliabilities. To examine interrater reliability, participants were assessed by two raters concurrently. To examine test–retest reliability, participants were re-assessed by the same rater up to 14 days after the original assessment (*M* ± *SD* = 6.1 ± 3.9 days). Each session took approximately 2 hours to complete (nb. owing to Covid-19 restrictions, a proportion of participants (28.9%) completed the MHS interview online via Teams/Zoom; all participants involved in interrater and test–retest sessions however participated in person). The study was performed in accordance with the Declaration of Helsinki (World Medical Association, 2013) and received ethics approval from the Swinburne University of Technology Human Research Ethics Committee (#20191226-1344). Participants also provided written, informed consent.

### Materials

#### The Multisensory Hallucinations Schedule

The MHS aimed to offer transdiagnostic assessment of the presence and characteristics of hallucinations across a range of sensory domains as well as prominent delusional themes. Its predecessor measure – the QPE – was a product of an International Consortium on Hallucination Research (ICHR) working group (Rossell et al., 2019). The MHS represents a major revision and expansion of this initial endeavour and comprises eight modules, assessing *A. Auditory hallucinations*, *V. Visual hallucinations*, *O. Olfactory hallucinations*, *T. Somatic-tactile hallucinations*, *G. Gustatory hallucinations*, *H. Other modalities of hallucinations*, *M. Multimodal hallucinations* and *D. Delusions*. Modules A, V, H and D were drawn from the QPE with modifications to content and structure, whereas the remaining modules were newly developed. Scale items were retained, modified/shifted or added/omitted based on expert consultation with the research team alongside lived experience input (see section ‘Lived experience involvement’). Response options were mostly standardised to align with 6-point Likert-type scales, with different wording employed for descriptive anchors across sensory modalities, as appropriate. These changes were enacted to improve administration following practised use of the QPE. For further information on content and structure revisions as well as scale definitions, format and scoring, refer to Table A and the full MHS interview in Supplementary materials.

In sum, MHS questions, organised by module, comprise a mix of items enquiring about descriptive phenomenology (including ~5% open-ended) as well as quantitative indicators. Administration commences with a single screening question per module; an affirmative response necessitates completion of the remaining module; otherwise, the interviewer is directed to the next module. Two time frames are typically assessed: lifetime (‘worst ever’ episode) and current (past 7 days); all participants complete the former, but only participants with current symptoms complete the latter. Scoring involves summation of all quantitative items within each module, with qualitative responses omitted (e.g. *description, complexity, location*, *personification*). Quantitatively, there are eight subscales: *Auditory* (12 items), *Visual* (9 items), *Olfactory* (10 items), *Somatic-tactile* (9 items), *Gustatory* (9 items), *Other modalities* (5 items), *Multimodal* (4 items) and *Delusions* (18 items). Administration time may range from 30 to 120 minutes, depending on number of modules endorsed.

##### Lived experience involvement

Active lived experience involvement was harnessed by consultation with an established lived experience advisory panel (LEAP) affiliated with the university, who regularly meet to discuss and advise on new research projects. A single 2-hour session was attended by four LEAP members, alongside four personnel from the research team. Prior to the consultation, a draft copy of the MHS was circulated for interested parties to peruse. On the day, a structured discussion was held, where a series of general questions were put forward by the research team covering each section of the proposed MHS, for instance including terminology/wording employed, items perceived to be helpful or misleading as well as suggestions for content additions and omissions. Towards the end, the floor was opened for questions and further nuanced discussion on related topics of interest. Upon conclusion, LEAP members were invited to get in touch with the research team to provide further feedback or input within a constrained (2-week) time frame. The research team then held a subsequent meeting to incorporate as many LEAP suggestions as practicable within the revised MHS.

#### Other measures

A standard demographic-clinical record elicited personal and mental health information. Several validated instruments were also administered, including the Depression Anxiety Stress Scales (DASS; Lovibond and Lovibond, 1995), Montgomery Asberg Depression Rating Scale (MADRS; Montgomery and Asberg, 1979), MUSEQ (Mitchell et al., 2017), PANSS (Kay et al., 1987), PSAS (de Chazeron et al., 2015), SAPS (Andreasen, 1984) and Young Mania Rating Scale (YMRS; Young et al., 1978). These measures comprised self-report (DASS, MUSEQ) and researcher-rated (MADRS, PANSS, PSAS, SAPS, YMRS) formats, aimed to tap into a range of hallucinations and delusions (MUSEQ, PANSS, PSAS, SAPS), as well as general psychopathology symptoms encountered in many psychiatric disorders (DASS, MADRS, SAPS, YMRS).

### Statistical analyses

Data analyses were performed using IBM SPSS Statistics, v.27. Basic demographic-clinical characterisation of participants was provided. In view of prevailing symptom-based approaches (Ford et al., 2014), subsequent pilot validation analysis was conducted across the entire cohort. This comprised three components: (1) confirmation of factor structure, (2) acceptability, convergent and discriminant validities, as well as (3) interrater and test–retest reliabilities and internal consistency.

Factor structure was verified via a series of principal component analysis (PCA) for each MHS subscale across the two time frames under consideration. Studies have shown that PCA using small sample sizes (i.e. *n* < 50) can yield high-quality results, even in the presence of data distortions (de Winter et al., 2009; Mundfrom et al., 2005). We thus opted to proceed with PCA for each MHS subscale where *n* > 30, where all items (except for descriptive and ranking questions) were submitted for analysis. Data suitability was assessed by checking that Kaiser–Meyer–Olkin > .6 (Kaiser, 1974) and Barlett’s (1954) test of sphericity was significant. Each PCA was conducted in a two-step process. In the first step, exploratory factor analysis was performed using Direct Oblimin rotation. Consensus solution for number of components was determined by: (1) considering eigenvalues > 1.0 and factor loadings > 0.4 (Hair et al., 2010), (2) examining screeplots (Cattell, 1966) and (iii) parallel analysis, assessing against corresponding criterion values for a randomly generated data matrix (Horn, 1965). Where there was discordance among these methods, the solution yielding the smallest number of meaningful factors was chosen. In the second step, confirmatory factor analysis was run by stipulating the previously determined number of factors. Three-factor models had been derived for *Auditory* and *Visual* subscales and a one-factor model for the *Delusions* subscale of the QPE (Rossell et al., 2019). We thus sought to examine if the same number of components would emerge in the current analysis.

Acceptability was assessed by calculating the MHS completion rate, with >95% considered satisfactory. To examine convergent validity, interscale correlations were performed between MHS subscales and relevant clinical instruments measuring equivalent constructs involving hallucinations and delusions (i.e. MUSEQ, PANSS, PSAS, SAPS), where moderate to high positive correlations were expected. For instance, Module A of the MHS was correlated with the *Auditory hallucinations* subscales of the MUSEQ and PSAS, as well as Item P3 of the PANSS; where modality-specific subscales were lacking, general hallucination scores were employed. To examine discriminant validity, interscale correlations were again performed, this time between MHS subscales and instruments measuring other forms of psychopathology (i.e. DASS, MADRS, YMRS), where low to moderate correlations were expected. All interscale correlations were performed using Kendall’s τ due to non-normality/ordinality.

To examine interrater reliability, equivalence testing was performed using intraclass correlation coefficients (ICCs; two-way mixed, absolute agreement, average measures) between ratings for the same participants across different researchers. To examine test–retest reliability, stability testing was performed, again using ICC, this time between ratings made by the same researcher across two time frames. Values of ICC < 0.5, 0.5 < ICC < 0.75, 0.75 < ICC < 0.9 and ICC > 0.90 are indicative of poor, moderate, good and excellent reliability, respectively (Koo and Li, 2016). To assess for internal consistency, Cronbach’s α was calculated for each MHS subscale across the two time frames, where Cronbach’s α = 0.6–0.7 is believed to reflect acceptable internal consistency (Hair et al., 2010). Intrascale correlations between MHS subscales were also performed, again using Kendall’s τ due to non-normality, with a conservative alpha set at *p* < 0.05/8 = 0.006 following Bonferroni correction.

## Results

### Participants

A total of 76 participants took part in the pilot validation study. Basic demographic-clinical information is shown in Table 1. All participants had a primary diagnosis of a schizophrenia spectrum disorder, mood disorder with psychotic features or other disorder with prominent hallucination symptoms (i.e. post-traumatic stress disorder or borderline personality disorder). Across the presented diagnoses, participants were relatively well-matched on most demographic-clinical variables (although no statistical groupwise comparisons were performed), with the exception of sex, where all participants in the Other disorders category were female. Figure 1 shows the frequency of endorsement of hallucinations (by modality) and delusions over the lifetime and current time frames. An adequate spread of hallucination modalities was demonstrated, with relatively higher endorsement of lifetime versus current experiences.

**Table 1.** Basic demographic and clinical information.

	Mean ± standard deviation or percentage		
	Schizophrenia spectrum disorders (*n* = 39)	Mood disorders with psychotic features (*n* = 30)	Other disorders with prominent hallucinations (*n* = 7)^a^
Age (years)	43.2 ± 9.9	41.3 ± 11.6	42.3 ± 16.4
Sex (% female)	61.5	56.7	100.0
Education (years)	15.6 ± 4.7	16.1 ± 4.0	14.7 ± 5.8
Marital status (% partnered)	23.1	10.0	28.6
Employment (% working)	46.2	46.7	42.9
Psychiatric comorbidity (% yes)	64.1	73.3	71.4
Neurological comorbidity (% yes)	38.5	40.0	57.1
Psychiatric medication (% yes)	100.0	86.7	71.4

Psychiatric comorbidities primarily related to anxiety and depressive disorders; neurological comorbidities included autism spectrum disorders, epilepsy and migraine; psychiatric medications typically referred to antipsychotics, antidepressants and mood stabilisers.

a Refers to participants with a post-traumatic stress disorder or borderline personality disorder diagnosis.

**Figure 1.**
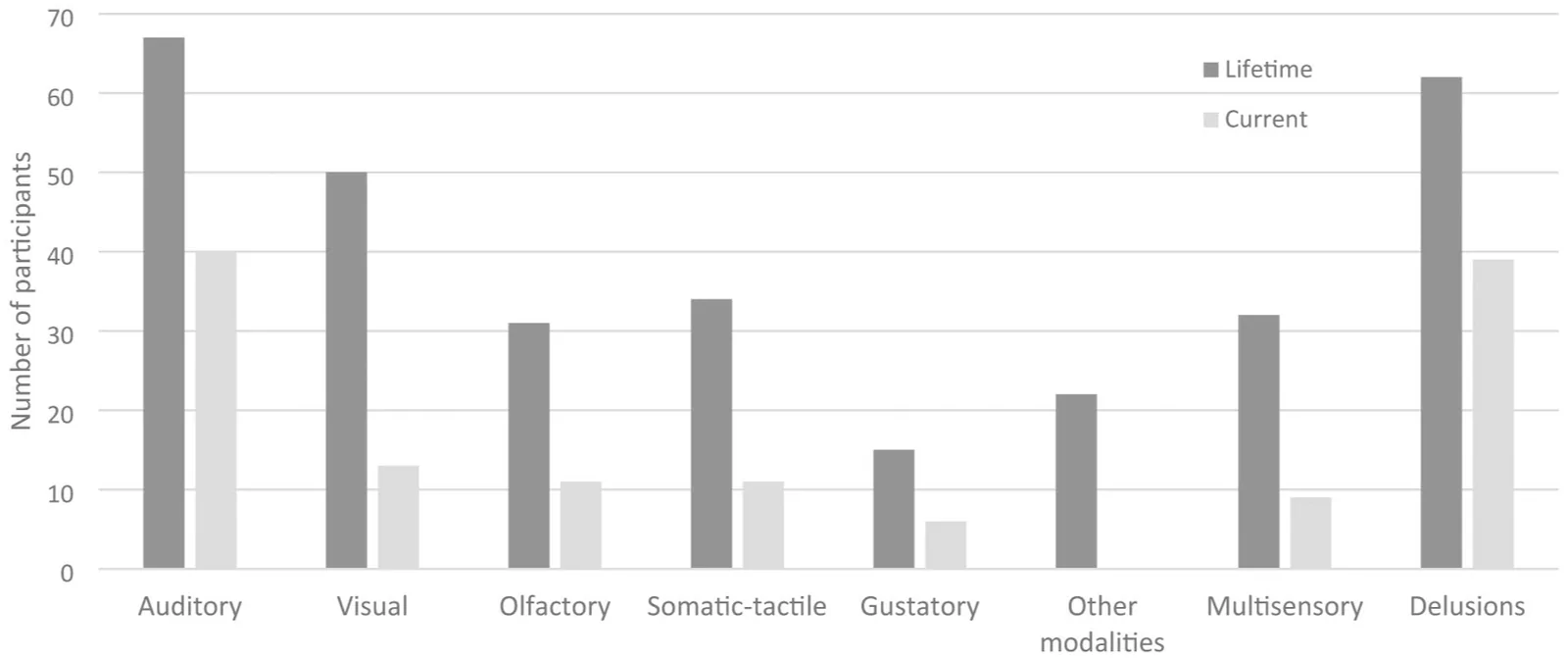
Frequency of endorsement of hallucinations (by modality) and delusions over the lifetime and current time frames (*N* = 76).

### Confirmation of factor structure

Eight MHS subscales had sufficient participant responses (see Figure 1) to be eligible for PCA. Table 2 shows these results, detailing items analysed per subscale, as well as their pattern/structure coefficients and communalities. In addition, Table A in Supplementary materials displays suitability data, parallel analysis results and component correlations, whereas Figure A in Supplementary materials illustrates scree plots used to aid determination of factor solutions.

**Table 2.** Principal components analysis, Direct Oblimin rotation for eligible MHS subscales (lifetime and current).

**Auditory lifetime – 43.4% variance explained**			
Item	Pattern coefficients (components 1, 2)	Structure coefficients (components 1, 2)	Communalities
A14 Impact	**0.823**, −0.147	**0.810**, −0.076	0.678
A15 Commenting	**0.807**, −0.050	**0.802**, 0.019	0.646
A13 Distress	**0.780**, 0.092	**0.788**, 0.159	0.630
A1 Frequency	**0.693**, 0.128	**0.704**, 0.188	0.512
A9 Compliance	**0.668**, −0.396	**0.634**, −0.388	0.558
A12 Valence	**0.616**, −0.130	**0.604**, −0.077	0.382
A3 Duration	**0.613**, 0.102	**0.622**, 0.155	0.397
A8 Interaction	**0.588**, 0.064	**0.594**, 0.155	0.357
A5 Complexity	**0.561**, **0.462**	**0.601**, **0.510**	0.573
A10 Personification	**0.458**, 0.363	**0.489**, **0.403**	0.370
A7 Conviction	0.260, −0.209	0.242, −0.186	0.102
A6 Location	−0.177, **0.611**	−-0.124, **0.596**	0.386
A16 Conversing	0.354, **0.525**	**0.400**, **0.556**	0.433
A11 Controllability	0.039, −**0.484**	−0.002, −**0.480**	0.232
A4 Intensity	0.309, 0.379	0.342, **0.406**	0.259
**Visual lifetime – 66.1% variance explained**			
Item	Pattern coefficients (components 1, 2, 3, 4)	Structure coefficients (components 1, 2, 3, 4)	Communalities
V13 Impact	**0.766**, 0.028, 0.368, 0.020	**0.797**, −0.059, **0.441**, −0.123	0.769
V1 Frequency	**0.761**, −0.124, −0.031, 0.117	**0.750**, −0.191, 0.045, 0.003	0.593
V3 Duration	**0.729**, 0.173, −0.127, −0.100	**0.718**, 0.122, −0.059, −0.215	0.574
V8 Interaction	**0.504**, −0.133, −0.293, −**0.469**	**0.561**, −0.143, −0.209, −**0.528**	0.610
V12 Distress	**0.486**, −0.109, **0.408**, −0.146	**0.559**, −0.169, **0.470**, −0.242	0.517
V7 Conviction	**0.487**, **0.763**, −0.119, 0.128	0.390, **0.724**, −0.121, 0.031	0.761
V4 Intensity	−0.006, **0.711**, 0.185, −0.095	−0.034, **0.705**, 0.149, −0.128	0.541
V10 Controllability	−0.247, **0.638**, −0.070, −0.057	−0.299, **0.665**, −0.128, −0.036	0.509
V9 Personification	00.59, −0.001, **0.849**, 0.304	0.095, −0.065, **0.838**, 0.248	0.793
V5 Complexity	−0.093, 0.075, **0.803**, −0.374	0.040, 0.050, **0.810**, −**0.407**	0.803
V6 Location	0.163, −0.199, 0.041, **0.847**	0.049, −0.244, 0.021, **0.825**	0.756
V11 Valence	0.323, −0.184, 0.221, −**0.645**	**0.463**, −0.202, 0.299, −**0.702**	0.706
**Olfactory lifetime – 56.5% variance explained**			
Item	Pattern coefficients (components 1, 2, 3)	Structure coefficients (components 1, 2, 3)	Communalities
O7 Conviction	**0.786**, 0.011, 0.157	**0.788**, 0.087, 0.159	0.645
O13 Impact	**0.745**, −0.050, −**0.450**	**0.738**, 0.037, −**0.446**	0.748
O1 Frequency	**0.699**, −0.141, −0.207	**0.683**, −0.064, −0.201	0.528
O4 Intensity	**0.608**, **0.328**, 0.104	**0.642**, 0.387, 0.098	0.527
O3 Duration	**0.566**, **0.403**, 0.215	**0.608**, **0.455**, 0.207	0.572
O10 Controllability	**0.548**, −0.284, **0.476**	**0.520**, −0.241, **0.485**	0.584
O5 Complexity	0.098, **0.754**, 0.006	0.174, **0.764**, −0.014	0.593
O11 Valence	−0.161, **0.665**, −**0.411**	−0.094, **0.660**, −**0.429**	0.631
O6 Location	−0.283, **0.649**, 0.212	−0.216, **0.615**, 0.194	0.501
O8 Interaction	0.215, **0.549**, 0.075	0.271, **0.569**, 0.062	0.375
O12 Distress	**0.424**, **0.511**, −**0.467**	**0.474**, **0.567**, −**0.479**	0.715
O9 Personification	0.027, −0.037, −**0.805**	0.020, −0.013, −**0.803**	0.647
O14 Self-smells	0.003, 0.073, **0.518**	0.012, 0.060, **0.516**	0.272
**Somatic-tactile lifetime – 72.7% variance explained**			
Item	Pattern coefficients (components 1, 2, 3, 4)	Structure coefficients (components 1, 2, 3, 4)	Communalities
T12 Distress	**0.842**, −0.215, −0.249, 0.009	**0.822**, −0.287, −0.303, 00.14	0.875
T11 Valence	**0.768**, 0.084, −0.148, 0.061	**0.744**, 0.031, −0.198, −0.109	0.633
T4 Intensity	**0.698**, 0.077, **0.424**, −0.063	**0.667**, 0.025, 0.379, −0.118	0.635
T13 Impact	**0.683**, −0.399, −0.043, 0.390	**0.697**, −**0.469**, −0.093, **0.421**	0.803
T1 Frequency	0.012, −**0.872**, −0.098, −0.040	0.095, −**0.865**, −0.091, 0.120	0.759
T3 Duration	0.341, −**0.851**, −0.059, −0.072	**0.422**, −**0.866**, −0.073, 0.068	0.880
T10 Controllability	0.380, **0.606**, −0.370, 0.003	0.352, **0.576**, −**0.400**, −0.117	0.630
T9 Personification	−0.012, −0.106, **0.845**, −0.022	−0.058, −0.108, **0.847**, −0.020	0.729
T8 Interaction	0.180, −0.069, −**0.672**, −0.153	0.237, −0.052, −**0.680**, −0.135	0.524
T5 Complexity	0.374, 0.354, **0.573**, −**0.492**	0.329, **0.407**, **0.556**, −**0.586**	0.874
T6 Location	−0.042, 0.348, −0.035, **0.935**	−0.113, 0.182, −0.054, **0.874**	0.887
T7 Conviction	0.174, −0.249, 0.255, **0.549**	0.153, −0.365, 0.234, **0.581**	0.496
**Multimodal lifetime – 76.9% variance explained**			
Item	Pattern coefficients (components 1, 2, 3)	Structure coefficients (components 1, 2, 3)	Communalities
M11 Impact	**0.933**, 0.157, 0.013	**0.917**, 0.054, 0.104	0.865
M9 Distress	**0.862**, −0.146, −0.169	**0.859**, −0.228, −0.064	0.785
M6 Preoccupation	**0.765**, 0.018, 0.105	**0.775**, −0.073, 0.188	0.612
M1 Presence	0.109, **0.897**, −0.036	0.007, **0.887**, −0.091	0.800
M5 Thematic content	−0.014, **0.833**, 0.192	−0.084, **0.821**, 0.128	0.710
M4 Temporal ordering	−0.177, **0.626**, −**0.441**	−0.294, **0.679**, −**0.507**	0.701
M7 Conviction	−0.042, 0.055, **0.963**	0.058, −0.013, **0.954**	0.915
**Delusions lifetime – 48.2% variance explained**			
Item	Pattern coefficients (components 1, 2, 3)	Structure coefficients (components 1, 2, 3)	Communalities
D5 Religiosity	**0.848**, −0.029, −0.030	**0.850**, −0.217, 0.117	0.723
D6 Grandeur	**0.778**, 0.1510., 0.047	**0.750**, −0.039, 0.142	0.584
D3 Guilt-sin	**0.696**, −0.031, 0.045	**0.710**, −0.202, 0.167	0.508
D2 Reference	**0.613**, −0.134, 0.186	**0.675**, −0.318, 0.318	0.516
D8 Nihilism	**0.431**, −0.143, −0.040	**0.458**, −0.233, 0.063	0.228
D9 Misidentification	0.381, −0.168, 0.234	**0.459**, −0.309, 0.335	0.305
D17 Distress	−0.295, −**0.923**, 0.051	−0.073, −**0.866**, 0.211	0.832
D18 Impact	0.116, −**0.783**, 0.015	0.299, −**0.813**, 0.211	0.675
D15 Preoccupation	0.036, −**0.723**, −0.074	0.191, −**0.715**, 0.095	0.517
D1 Paranoia	0.154, −**0.706**, 0.182	0.347, −**0.782**, 0.367	0.672
D16 Conviction	0.142, −**0.675**, −0.104	0.280, −**0.684**, 0.072	0.494
D13 Thought insertion	0.191, 0.113, **0.748**	0.289, −0.100, **0.754**	0.608
D4 Control	−0.036, −0.028, **0.653**	0.079, −0.167, **0.653**	0.428
D11 Mind-reading	0.128, −0.095, **0.626**	0.253, −0.266, **0.669**	0.476
D14 Thought withdrawal	−0.078, 0.159, **0.535**	−0.026, 0.056, **0.486**	0.271
D7 Somatic	0.148, 0.007, **0.535**	0.235, −0.148, **0.558**	0.332
D12 Thought broadcast	−0.314, −0.161, **0.427**	−0.206, −0.185, **0.412**	0.270
D10 Jealousy	0.097, −0.159, **0.402**	0.200, −0.272, **0.454**	0.245
**Auditory current – 39.2% variance explained**			
Item	Pattern coefficients (components 1, 2)	Structure coefficients (components 1, 2)	Communalities
A15 Commenting	**0.762**, 0.224	**0.792**, 0.325	0.677
A5 Complexity	**0.722**, 0.174	**0.745**, 0.270	0.585
A3 Duration	**0.656**, 0.154	**0.677**, 0.241	0.481
A8 Interaction	**0.647**, −0.218	**0.618**, −0.131	0.429
A16 Conversing	**0.634**, 0.166	**0.657**, 0.251	0.458
A1 Frequency	**0.593**, −0.250	**0.559**, −0.171	0.375
A14 Impact	**0.513**, 0.254	**0.547**, 0.322	0.362
A9 Compliance	**0.461**, −0.235	**0.430**, −0.173	0.239
A10 Personification	0.344, 0.298	0.384, 0.344	0.234
A11 Controllability	0.089, −0.002	0.089, 0.009	0.008
A13 Distress	0.139, **0.747**	0.239, **0.766**	0.606
A12 Valence	0.190, **0.712**	0.285, **0.738**	0.580
A4 Intensity	−0.286, **0.690**	−0.194, **0.652**	0.506
A6 Location	−0.079, **0.553**	−0.005, **0.543**	0.301
A7 Conviction	−0.079, −0.172	−0.102, −0.183	0.040
**Delusions current – 44.6% variance explained**			
Item	Pattern coefficients (components 1, 2)	Structure coefficients (components 1, 2)	Communalities
D13 Thought insertion	**0.760**, −**0.465**	**0.755**, −**0.457**	0.787
D2 Reference	**0.743**, −0.115	**0.742**, −0.106	0.564
D18 Impact	**0.728**, 0.274	**0.731**, 0.282	0.609
D11 Mind-reading	**0.694**, −0.255	**0.691**, −0.248	0.543
D9 Misidentification	**0.686**, −0.547	**0.680**, −**0.540**	0.763
D4 Control	**0.669**, 0.077	**0.669**, 0.085	0.454
D1 Paranoia	**0.661**, 0.294	**0.664**, 0.301	0.527
D15 Preoccupation	**0.628**, 0.280	**0.631**, 0.287	0.476
D17 Distress	**0.600**, 0.355	**0.604**, 0.362	0.491
D10 Jealousy	**0.596**, −0.142	**0.594**, −0.136	0.373
D16 Conviction	**0.559**, 0.391	**0.563**, 0.397	0.470
D5 Religiosity	**0.494**, 0.369	**0.498**, 0.374	0.384
D12 Thought broadcast	**0.457**, −0.368	**0.453**, −0.363	0.341
D7 Somatic	**0.436**, −0.389	**0.431**, −0.384	0.338
D8 Nihilism	0.382, −0.072	0.381, −0.068	0.150
D6 Grandeur	0.234, **0.500**	0.239, **0.502**	0.307
D14 Thought withdrawal	0.245, −**0.409**	0.241, −**0.406**	0.225
D3 Guilt-sin	0.319, 0.340	0.323, 0.343	0.220

MHS: Multisensory Hallucinations Schedule. All MHS items were entered into the corresponding principal components analysis by subscale, except for descriptive or ranking items (e.g. A2, V2, O2, M12, M13), which were omitted as they were more qualitative in nature. Only MHS subscales meeting minimum recommended sample size requirements were analysed. Factor loadings > 0.4 denoted in bold.

The total variance explained by MHS subscales ranged from 39.2% to 76.9%, and the number of factor solutions identified varied from two to four components. High component loadings were largely observed throughout, with most variables loading substantially onto a single component. Moderate to high communality values (>0.3) were also recorded, denoting most items fit well within their respective subscale. A few exceptions existed, namely, *Conviction*, which failed to load onto *Auditory lifetime; Personification*, *Controllability* and *Conviction*, which failed to load onto *Auditory current*; as well as *Nihilism* and *Guilt-Sin*, which failed to load onto *Delusions current*.

Factor naming^1^ (with components listed in paratheses) involved: *Emotional* (1) and *Perceptual* (2) for *Auditory lifetime; Perceptual* (1) and *Emotional* (2) for *Auditory current; Emotional* (1), *Cognitive* (2), *Identity* (3) and *Perceptual* (4) for *Visual lifetime; Emotional* (1), *Perceptual* (2) and *Identity* (3) for *Olfactory lifetime; Emotional* (1), *Perceptual* (2), *Identity* (3) and *Cognitive* (4) for *Somatic-tactile lifetime; Emotional* (1), *Content* (2) and *Cognitive* (3) for *Multimodal lifetime; Content* (1), *Impact* (2) and *Agency* (3) for *Delusions lifetime*; and *Impact* (1) and *Identity* (2) for *Delusions current*.

### Acceptability, convergent and discriminant validities

Only a single participant dropped out midway, with the remaining 98.7% completing the MHS. This shows that majority of participants found the measure to be acceptable. Convergent and discriminant validities are shown in Table 3. Significant high-positive correlations were largely observed for MHS subscales and corresponding items/subscales of convergent measures; whereas low to moderate correlations were recorded for MHS subscales and total scores of divergent measures, most of which were non-significant.

**Table 3.** Validity statistics.

Other measures	MHS subscales (lifetime, current)							
	Auditory	Visual	Olfactory	Somatic-tactile	Gustatory	Other modalities	Multimodal	Delusions
***Convergent***								
*MUSEQ (A, V, O,* *G, BS)*	0.291**, 0.392**	0.410**, 0.439**	0.441**, 0.339**	0.393**, 0.240*	0.415**, 0.328**	-	-	-
*PANSS (P1, P3)*	0.359**, 0.696**	0.280**, 0.276**	0.202*, 0.158	0.209*, 0.276**	0.092, 0.083	0.045, -	0.210*, 0.323**	0.186, 0.439**
*PSAS (A, V, G/O, C)* ^a^	−0.021, 0.575**	0.287, 0.489*	0.156, 0.716**	0.471*, 0.656**	0.306, 0.533**	-	-	-
*SAPS (H, D)*	0.658**, 0.612**	0.676**, 0.656**	0.759**, 0.244	0.807**, 0.777**	0.120, 0.211*	0.184, -	0.384**, 0.306**	0.654**, 0.653**
***Divergent***								
*DASS*	0.124, 0.233*	0.170*, 0.315**	−0.057, 0.052	0.181*, 0.159	0.104, 0.260**	0.099, -	0.086, 0.181	0.019, 0.257**
*MADRS* ^a^	−0.219, 0.005	−0.066, 0.324	0.480**, 0.056	0.208, 0.204	0.014, 0.241	−0.174, -	−0.220, 0.281	−0.127, 0.150
*YMRS*	0.111, 0.129	0.211*, 0.133	−0.131, −0.174	−0.011, 0.031	−0.188, −0.198*	−0.073, -	0.143, 0.112	−0.023, 0.085

MHS: Multisensory Hallucinations Schedule; MUSEQ: Multi-Modality Unusual Sensory Experiences Questionnaire (A = Auditory, V = Visual, O = Olfactory, G = Gustatory, BS = Bodily sensations); PANSS: Positive and Negative Syndrome Scale (P1 = Delusions, P3 = Hallucinatory behaviour); PSAS: Psycho-Sensory Hallucinations Scale (A = Auditory, V = Visual, G/O = Gustatory/olfactory, C = Cenesthetic); SAPS: Scale for the Assessment of Positive Symptoms (H = Hallucinations, D = Delusions); DASS: Depression Anxiety Stress Scales; MADRS: Montgomery Asberg Depression Rating Scale; YMRS: Young Mania Rating Scale. For the purpose of the current study, corresponding individual item or subscale scores for the MUSEQ (Auditory, Visual, Olfactory, Gustatory, Bodily sensations subscales), PANSS (P1 Delusions and P3 Hallucinatory behaviour items), PSAS (Auditory, Visual, Olfactory/gustatory, Cenesthetic subscales) and SAPS (Hallucinations, Delusions subscales) were used to perform interscale correlations with the relevant MHS subscales, with general hallucination subscale scores employed in cases where modality-specific subscales did not exist. Total scores for the DASS, MADRS and YMRS were utilised. No participants endorsed *Other modalities* within the current time frame; no validity statistics could be computed.

a Missing data was noted, where *n* = 21.

* *p* < 0.05, **p < 0.001.

### Interrater and test–retest reliabilities and internal consistency

Reliability statistics are presented in Table 4, where it is noted that the ICC for equivalence exhibited mostly moderate to high values for lifetime (0.831–0.981) and current (0.574–0.993, except for *Multimodal*) time frames. Similarly, ICC for stability showed largely moderate to high values for lifetime (0.776–0.980) and current (0.535–0.991, except for *Multimodal*) time frames. The MHS subscales also demonstrated acceptable to excellent internal consistencies for lifetime (Cronbach’s α = 0.576–0.817) and current (Cronbach’s α = 0.462–0.851) time frames. No participants endorsed *Other modalities current* meaning no equivalence, stability or internal consistency statistics could be computed for this subscale. Intrascale MHS correlations are also presented in Table 4, where some significant positive correlations were found among the unimodal subscales across different time frames. The multimodal (and delusions, to a lesser extent) subscale(s) also showed patterns of significant positive correlations with unimodal subscales. Remaining intrascale correlations showed low positive values, but were non-significant.

**Table 4.** Reliability statistics and intrascale correlations.

MHS subscales	Equivalence (ICC)		Stability (ICC)			Internal consistency (Cronbach’s α)	
	Lifetime	Current	Lifetime		Current	Lifetime	Current
***Auditory***	0.878	0.903	0.900		0.909	0.817	0.664
***Visual***	0.978	0.586	0.980		0.594	0.698	0.462
***Olfactory***	0.887	0.574	0.880		0.535	0.703	0.637
***Somatic-tactile***	0.922	0.812	0.934		0.817	0.730	0.583
***Gustatory***	0.981	0.993	0.977		0.991	0.659	0.484
***Other modalities***	0.831	-	0.776		-	0.576	-
***Multimodal***	0.925	0.376	0.900		0.253	0.684	0.751
***Delusions***	0.917	0.915	0.937		0.908	0.799	0.851
	Intrascale correlations (lifetime, current)						
	Auditory	Visual	Olfactory	Somatic-tactile	Gustatory	Other modalities	Multisensory
***Auditory***	1						
***Visual***	0.368*, 0.407*	1					
***Olfactory***	0.257*, 0.182	0.215, 0.299	1				
***Somatic-tactile***	0.348*, 0.212	0.231, 0.373*	0.236, 0.335*	1			
***Gustatory***	0.033, 0.023	0.034, 0.210	0.467*, 0.564*	0.273, 0.286	1		
***Other modalities***	0.203, -	0.128, -	0.101, -	0.198, -	0.115, -	1	
***Multimodal***	0.385*, 0.429*	0.558*, 0.696*	0.272*, 0.582*	0.372*, 0.445*	0.136, 0.263	0.227, -	1
***Delusions***	0.276*, 0.350*	0.221, 0.370*	0.183, 0.428*	0.267*, 0.263	0.075, 0.102	0.061, -	0.248*, 0.494*

MHS: Multisensory Hallucinations Schedule; ICC: intraclass correlation coefficients. No participants endorsed *Other modalities* within current time frame; no equivalence and stability statistics or intrascale correlations could be computed. Mean time lapse between test–retest = 6.1 ± 3.9 days (range, 1–14 days).

* Significant at *p* < 0.05/8 = 0.006 (two-tailed) following Bonferroni correction.

## Discussion

The current study aimed to perform pilot validation of a new hallucinations measure – the MHS, designed for comprehensive phenomenological assessment across multiple hallucination modalities in psychosis. Participant clinical-demographic variables across the primary diagnostic groups were largely similar, supporting our decision to conduct pilot validation analysis across the entire cohort.

Confirmation of factor structure by a series of PCA identified two to four components for each eligible MHS subscale. Despite limited participant numbers (*n* = 31–67), these factor solutions evidenced high component loadings, moderate to high communalities and were able to explain a significant proportion of variance (39.2–76.9%), with the greatest amount of variance explained by a three-factor solution. Only six (out of a total of 110) items failed to load onto their respective subscales (see section ‘Future directions’). Yet the existence of lower communality values may not signify that the constructs represented by these items do not belong on the scale. Rather these items may instead capture aspects of hallucinatory experiences unrelated to the other MHS items, but still imperative to a comprehensive assessment. Determination of which facets of hallucinations are most relevant to its accurate assessment is an ongoing process, invigorated by continued debate within the field.

There was broad consistency across factor names, especially for unimodal subscales, with two common labels *Emotional* and *Perceptual* for *Auditory* (*lifetime* and *current*), *Visual lifetime*, *Olfactory lifetime* and *Somatic-tactile lifetime*; a third shared label involving *Identity* for *Visual lifetime*, *Olfactory lifetime* and *Somatic-tactile lifetime*; and a fourth overlapping label involving *Cognitive* for *Visual lifetime* and *Somatic-tactile lifetime*. Different factor names were noted for the remaining subscales, with these deviations to be expected, likely owing to distinct item content within the *Multimodal* and *Delusions* subscales. Overall, there was respectively good and acceptable factor structure convergence for the *Auditory* and *Delusions* subscales across the two time frames considered. Our findings somewhat vary from the QPE scale validation (Rossell et al., 2019), in that one- to three-factor solutions were identified for their *Auditory*, *Visual* and *Delusions* subscales. Our failure to replicate these previous factor structures was however not surprising, given significant content and structure modifications made to the MHS. Yet we were able to explain a comparable (cf. 57.3–62.1%) proportion of variance within the models and also managed to broaden the number of sensory modalities assessed and validated.

The MHS demonstrated excellent acceptability, with a completion rate of 98.7%. Examination of convergent validities generally showed significant moderate positive correlations with parallel items/subscales of the MUSEQ, PANSS, PSAS and SAPS. Several exceptions did exist. The *Other modalities* subscale was not significantly associated with convergent measures employed, owing to a lack of existing instruments evaluating these constructs (e.g. kinaesthetic-vestibular, autoscopic, hypnagogic-hypnopompic hallucinations). Similarly, the *Gustatory* (and to a lesser extent *Olfactory*) subscale(s) did not correlate as well with the PANSS and SAPS, again likely because these instruments did not specifically tap into these sensory modalities. The MHS lifetime (but not current) subscales also showed lower correlations with the PSAS (as the latter was based on the past week only). Inspection of divergent validities likewise demonstrated mostly low to moderate non-significant correlations with total scores of the DASS, MADRS and YMRS. Isolated significant correlations were noted but expected, given known overlaps between hallucinations and general psychopathology symptoms.

Broadly, the MHS demonstrated good to excellent interrater and test–retest reliabilities across the two time frames considered, apart from *Visual current*, *Olfactory current* and *Multimodal current*, where poor to moderate interrater and test–retest reliabilities were noted, perhaps owing to limited participant numbers. Internal consistencies for the two time frames were also mostly acceptable, with the exception of *Other modalities lifetime*, *Visual current*, *Somatic-tactile current* and *Gustatory current*. Finally, intrascale correlations revealed low to moderate positive correlations (mostly non-significant), indicating that MHS subscales adequately tap into distinct hallucination and delusion experiences.

Direct comparisons of the psychometric properties of the MHS relative to existing instruments is not straightforward for a range of reasons. Some earlier tools such as the SAPS lacked psychometric validation (Andreasen, 1984), whereas others like the PSYRATS and MUPS were only able to recruit a small number of participants or assess limited components of their measure (Carter et al., 1995; Haddock et al., 1999). More recently developed assessments involving the PSAS, MUSEQ and QPE were able to offer more rigorous and sophisticated scale validation (de Chazeron et al., 2015; Mitchell et al., 2017; Rossell et al., 2019), but differing scale structures, methods of analyses and the numerous statistics generated nevertheless rendered meaningful comparisons challenging. Broadly, we may conclude that the acceptability, validities and reliabilities of the MHS appear at least comparable to existing measures in the field.

### Limitations

The current study was subject to several limitations. First, there was a lack of formalised methodology employed, such as a Delphi process, during our design phase. That being said, we were able to leverage off accumulated expert knowledge underlying initial design of the QPE, supplemented by active lived experience input. Moreover, the Delphi method is associated with known limitations, including potential lack of panel representativeness and time/resource inefficiencies (Nasa et al., 2021). Second, our pilot validation analysis was liable to certain methodological weakness, largely owing to relatively limited participant numbers. In particular, this affected our validation analyses for lesser known hallucination modalities (e.g. *Other modalities* and *Multimodal* subscales) and within the current time frame (where some MHS subscales were deemed not suitable for PCA). Yet this was balanced against demonstrated methodological strengths, including robust factor loadings and proportion of variance explained for eligible PCA, accompanied by use of a mix of researcher-rated and self-report instruments demonstrating good convergent and divergent validities. Acceptable to high reliabilities, involving interrater and test–retest reliabilities, and internal consistency, were also noted.

Our preliminary attempt at MHS scale validation has introduced benefits that outweigh possible downsides. For instance, this represents an initial foray into the construction and validation of an inclusive hallucinations assessment, especially tapping into lesser known sensory modalities. Despite the current focus on validation of quantitative items, the MHS is complemented by its offer of a mixture of open-ended and ranking qualitative questions, designed to furnish rich descriptive detail, beyond basic categorical responses. Furthermore, flexible administration of select modules allows a unique focus on specific variables of interest and offsets the demands of typical time-limited research environments.

### Future directions

Given this represents a preliminary validation attempt, much more replication work, preferably employing larger participant cohorts, remains to be done. In doing so, it is vital to ensure that appropriate participant numbers are achieved during assessment of lesser known hallucination modalities, especially within a current time frame. Instrument refinement could involve possible modification or removal of the small number of items (i.e. *Conviction*, *Controllability*, *Personification*, *Nihilism* and *Guilt-Sin*) that failed to load appropriately onto the factors identified. Moreover, visual and somatic-tactile (and to a lesser extent, olfactory) hallucinations appear to be more multifaceted in nature (characterised by up to four factors), relative to auditory hallucinations. This finding is notable and deserves further investigation. More broadly, future validation endeavours may look to recruit participants from other psychiatric and neurological groups (beyond psychotic illness) to document their multisensory hallucinatory experiences. This may be pertinent in neurological disorders, with its predominant focus on visual hallucinations (akin to the emphasis on auditory hallucinations in psychiatry), or even in populations experiencing unique stressors (e.g. bereavement or sleep disorders). Finally, alternate administration formats with necessary revisions, such as online or self-report, may be explored to maximise access and utility of the MHS.

## Conclusion

The current study comprehensively documented the design and pilot validation of the new MHS, aimed to inclusively assess hallucinations across a major range of sensory modalities. During the design phase, we were able to leverage off accumulated expert knowledge contained within its predecessor measure, also incorporating lived experience input. Our pilot validation analyses have demonstrated that the MHS has acceptable factor structure across its subscales, excellent acceptability as well as robust convergent and divergent validities. It has also shown acceptable to good reliabilities in the form of equivalence, stability and internal consistency. Pending further refinement and replication, this new tool may be deployed to help advance the field of hallucination studies in an inclusive manner.

## Supplemental Material

sj-docx-1-anp-10.1177_00048674261457016 – Supplemental material for The Multisensory Hallucinations Schedule to assess hallucinations across multiple sensory modalities in psychosis: A pilot validation study Supplemental material, sj-docx-1-anp-10.1177_00048674261457016 for The Multisensory Hallucinations Schedule to assess hallucinations across multiple sensory modalities in psychosis: A pilot validation study by Wei Lin Toh, Adrienne Bell, Mikaela Bere and Susan Lee Rossell in Australian & New Zealand Journal of Psychiatry

## Data Availability

The dataset is unsuitable to post, as it includes sensitive or confidential information, such as patient data. However, it is available on request by qualified researchers-scientists. Requests require a concept proposal describing the purpose of data access, appropriate ethical approval and provision for data security. All data analysis scripts and results files are available for review.

## Notes

1. Emotional = Affect-laden aspects of hallucinations; Perceptual = Related to physical characteristics of experiences; Cognitive = Associated with interpretation and sensing; Identity = Pertinent to the self or some other entity; Content = Involving subjective topics or themes; Impact = Bearing influence or consequence; Agency = Having autonomy or some degree of control.
