# Supplementary material for: The Multisensory Hallucinations Schedule to assess hallucinations across multiple sensory modalities in psychosis: A pilot validation study
Source: Aust N Z J Psychiatry. 2026 Jun 23;60(11):1074–88. doi: 10.1177/00048674261457016 (PMC13716980; doi:10.1177/00048674261457016)
Supplement: Supplementary file 1 [file sj-docx-1-anp-10.1177_00048674261457016.docx]

Table A

*Content and structure revisions as well as scale definitions, format and scoring of for the Multisensory Hallucinations Schedule (MHS)*

|  | **A. Auditory hallucinations** | **V. Visual hallucinations** | **O. Olfactory hallucinations** | **T. Somatic-tactile hallucinations** | **G. Gustatory hallucinations** | **H. Other modalities of hallucinations** | **M. Multimodal hallucinations** | **D. Delusions** |
| --- | --- | --- | --- | --- | --- | --- | --- | --- |
| **Scale definitions** | To assess the false perception of sensory experiences involved in hearing, seeing, smelling, touching and tasting as well as related physical, cognitive and emotional characteristics | | | | | To gauge experiences involving common modalities outside of the five main senses | To evaluate sensory experiences occurring concurrently in time and/or sharing thematic overlaps | To assess a range of themes involving false beliefs about external reality held despite incontrovertible evidence to the contrary |
| **Content and structure revisions** | ***Addition:*** Intensity, controllability, personification, voices commenting/conversing, global  ***Omission:*** Repetition, time of occurrence, auditory illusions  ***Modification:*** Musical hallucinations (moved to Module H) | ***Addition:*** Intensity, controllability, personification, global  ***Omission:*** Repetition, time of occurrence, compliance, visual illusions, passage hallucinations | ***Addition:*** Duration, intensity, complexity, location, conviction, interaction, personification, controllability, emotional valence, distress, impact, global; self-smells (for Module O only); frequency, description, age of onset, trauma-related (for Module G only) | | | ***Addition:*** Kinaesthetic-vestibular, autoscopic, hypnagogic-hypnopompic; frequency, description, sensory modality, context/triggers | ***Addition:*** Description, sensory modality, temporal ordering, thematic content, preoccupation, conviction, distress, impact | ***Addition:*** Jealousy, mind-reading, passivity phenomena  ***Omission:*** Delusions secondary to hallucinations  ***Modification:*** Preoccupation, conviction, distress, impact (single item each across delusions overall) |
| **Question format** | Mixture of one false-choice, two open-ended and 12-15 6-point Likert scale questions for Modules A, V, O, T and G; 16 4-7-point Likert scale questions for Module H; one false-choice, four open-ended, six 5-6-point Likert scale and two ranking questions for Module M; 18 6-point Likert scale questions for Module D | | | | | | | |
| ^ꝉ^ **Scoring**  **(items; range)** | A1+A3+A4+A7+A8+A9+A11+A12+A13+A14+A15+A16; 0-60 | V1+V3+V4+V7+V8+V10+V11+V12+V13;  0-45 | O1+O3+O4+O7+O8+O10+O11+O12+O13+  O14; 0-50 | T1+T3+T4+T7+T8+T10+T11+T12+T13;  0-45 | G1+G3+G4+G7+G8+G10+G11+G12+  G13; 0-45 | H1+H4+H7+  H11+H16;  0-25 | M6+M7+M9+M11; 0-20 | D1+D2+D3+D4+D5+D6+D7+D8+D9+D10+D11+D12+D13+  D14+D15+D16+D17+D18; 0-90 |

*Note.* ^ꝉ^ Scoring involves summation of all quantitative items within each module, with qualitative responses omitted (e.g. *description, complexity, location*, *personification*). Higher scores denote greater symptom severity per modality.

Table B

*Data suitability, parallel analysis and components correlations for principal components analysis*

|  | ***Data suitability*** | | ***Parallel analysis*** | | | ***Component correlations*** |
| --- | --- | --- | --- | --- | --- | --- |
|  | *Kaiser-Meyer-Olkin* | *Barlett’s test of sphericity (χ^2^, p)* | *Component* | *Initial eigenvalues* | *Random eigenvalues* | *r* |
| ***Auditory lifetime***  ***Visual lifetime***  ***Olfactory lifetime***  ***Somatic-tactile lifetime***  ***Multimodal lifetime***  ***Delusions lifetime***  ***Auditory current***  ***Delusions current*** | .780  .570  .437  .471  .454  .732  .504  .541 | 328.9, <.001  153.6, <.001  110.2, .010  132.3, <.001  79.4, <.001  362.1, <.001  194.6, <.001  399.8, <.001 | **1**, **2**, 3  **1**, **2**, **3**, **4**, 5  **1**, **2**, **3**, 4  **1**, **2**, **3**, **4**  **1, 2**, **3**  **1**, **2**, **3**, 4  **1**, **2**, **3**, **4**, **5**, 6  **1**, **2**, 3 | 4.936, 1.580, 1.367  3.154, 1.765, 1.640, 1.375,1.056  3.369, 2.202, 1.767, 1.199  3.195, 2.196, 1.966, 1.370  2.404, 1.833, 1.148  4.642, 2.186, 1.857, 1.304  3.864, 2.015, 1.532, 1.382, 1.341, 1.082  5.923, 2.099, 1.483 | 1.741, 1.549, 1.416  1.605, 1.440, 1.311, 1.205, 1.107  1.680, 1.486, 1.344, 1.244  1.617, 1.440, 1.318, 1.202  1.287, 1.221, 1.093  1.824, 1.647, 1.517, 1.411  1.719, 1.546, 1.418, 1.299, 1.205, 1.118  1.820, 1.647, 1.517 | .086  -.159–.099  -.026–.102  -.182–-.008  -.110–-.075  -.231–.165  .133  .011 |

*Note.* Only initial eigenvalue>1 listed; random eigenvalues generated by the Monte Carlo Principal Components Analysis for Parallel Analysis, with number of components identified highlighted in bold (the five-factor solution generated for the *Auditory current* subscale was ultimately rejected in lieu of a more succinct two-factor solution supported by the corresponding scree plot. Remaining MHS subscales (not listed here) deemed not appropriate for principal components analysis, owing to insufficient participant numbers (*n*<30).

*Figure A.* Exploratory principal components analysis scree plots for MHS subscales (from top to bottom and left to right): Auditory lifetime, Visual lifetime, Olfactory lifetime, Somatic-tactile lifetime, Multimodal lifetime, Delusions lifetime, Auditory current and Delusions current.
